# Investigation of FoxO3 dynamics during erythroblast development in β-thalassemia major

**DOI:** 10.1371/journal.pone.0187610

**Published:** 2017-11-03

**Authors:** Naruchit Thanuthanakhun, Lalana Nuntakarn, Somponnat Sampattavanich, Usanarat Anurathapan, Suphanun Phuphanitcharoenkun, Savichaya Pornpaiboonstid, Suparerk Borwornpinyo, Suradej Hongeng

**Affiliations:** 1 Department of Biotechnology, Faculty of Science, Mahidol University, Bangkok, Thailand; 2 Department of Obstetrics and Gynecology, Faculty of Medicine, Ramathibodi Hospital, Mahidol University, Bangkok, Thailand; 3 Siriraj Laboratory for Systems Pharmacology, Department of Pharmacology, Faculty of Medicine, Siriraj Hospital, Mahidol University, Bangkok, Thailand; 4 Department of Pediatrics, Faculty of Medicine, Ramathibodi Hospital, Mahidol University, Bangkok, Thailand; 5 Merck Ltd, Bangkok, Thailand; 6 Excellent Center for Drug Discovery, Faculty of Science, Mahidol University, Bangkok, Thailand; University of Washington, UNITED STATES

## Abstract

The FoxO3 transcription factor is a key regulator of oxidative stress and erythroid maturation during erythropoiesis. In this study, we explored the involvement of FoxO3 in severe β-thalassemia. Using primary CD34^+^ hematopoietic progenitor cells from patients with β-thalassemia major, we successfully developed an *in vitro* model of ineffective erythropoiesis. Based on this model, FoxO3 activity was quantified in single cells using high throughput imaging flow cytometry. This study revealed a significant reduction of FoxO3 activity during the late stage of erythroblast differentiation in β-thalassemia, in contrast to erythropoiesis in normal cells that maintain persistent activation of FoxO3. In agreement with the decreased FoxO3 activity in β-thalassemia, the expression of FoxO3 target genes was also found to decrease, concurrent with elevated phosphorylation of AKT, most clearly at the late stage of erythroid differentiation. Our findings provide further evidence for the involvement of FoxO3 during terminal erythropoiesis and confirm the modulation of the PI3K/AKT pathway as a potential therapeutic strategy for β-thalassemia.

## Introduction

β-Thalassemias are inherited monogenic disorders caused by mutations within the β-globin gene or other sites in rare cases [1]. This genetic defect results in a reduction or absence of normal β-globin synthesis, which leads to an imbalance of α- and β-globin chains [2]. The excessive free α-globin chains are unstable and can induce formation of reactive oxygen species (ROS) leading to damage of cellular compartments and promoting premature apoptosis of erythroid cells [2–4]. These pathophysiological events contribute to the arrest of erythroid maturation and reduction of red blood cell production, collectively defined as ineffective erythropoiesis [3, 5].

The molecular mechanisms underlying both normal and ineffective erythropoiesis have been extensively investigated with a prominent role identified for the Forkhead box O3 transcription factor (FoxO3). Previous studies have shown that FoxO3 is involved in the regulation of a wide range of activities crucial for maturation of erythroid cells. These include the oxidative stress response, cell cycle regulation, and enucleation processes [6–8]. FoxO3-deficient erythroid precursor cells in mice and zebrafish models exhibit elevated levels of ROS, increased oxidative damage, and decreased expression of genes involved in terminal erythroid maturation, resulting in shortened lifespan of red blood cells [7–10]. FoxO3 is negatively regulated by AKT (protein kinase B) through phosphorylation of Thr32, Ser253 and Ser315 in FoxO3, promoting nuclear-to-cytosolic translocation and preventing FoxO3 from interacting with its target genes [11]. Treatment of β-thalassemic mice with rapamycin (mTOR inhibitor) [10] or resveratrol [6] was found to enhance FoxO3 activity and improve erythroid maturation and survival. FoxO3 appears to be an important therapeutic target in patients with β-thalassemia, and the use of agents to restore the activity of FoxO3 is currently being investigated [12]. Even though much is known about the role of FoxO3, its regulation during erythroid differentiation in β-thalassemia patients is still poorly described.

This study examined how the activity of FoxO3 is altered during late erythropoiesis in patients with severe β-thalassemia. An *in vitro* model of ineffective erythropoiesis was prepared from CD34^+^ hematopoietic progenitor cells (CD34^+^ cells) isolated from patients with β-thalassemia major following *in vitro* differentiation using three-stage erythroid cultures. Changes in FoxO3 activity were monitored by quantifying its intracellular localization in single cells during different developmental stages of erythroblasts. Potential changes of AKT activity as well as the transcriptional changes of FoxO3 target genes were also investigated. Our data clarify the dynamics of FoxO3 activation and confirms the PI3K/AKT pathway as a promising therapeutic target in β-thalassemia.

## Materials and methods

### Patients, isolation of CD34^+^ cells and erythroid culture

Bone marrow samples were collected from four β-thalassemia major patients and leukapheresis products were collected from five normal donors at Ramathibodi Hospital, Mahidol University, Thailand. Three patients were diagnosed with β^0^-thalassemia/hemoglobin E and one with homozygous β^0^-thalassemia. The age of patients ranged from 2 to 9 years old. All patients had clinical symptoms of thalassemia major including thalassemic facies, hepatosplenomegaly, and hemoglobin levels of less than 7 g/dL without red blood cell transfusion at the time of diagnosis. All patients received transfusions every 3 to 4 weeks to maintain hemoglobin levels above 9 g/dL. Clinical characteristics of the patients are described in Table 1. Sample collection in this study was approved by the Ethical Committee on Human Rights Related to Research Involving Human Subjects at Ramathibodi Hospital. Written informed consent was obtained from legal guardians on behalf of all child patients and all normal donors in accordance with the Declaration of Helsinki.

**Table 1:** Clinical characteristics of patients.

Patient	Sex	Age	Serum ferritin (ng/mL)	Genotype (mutation)
1	M	2.5	1286.8	β^0^(IVS2-654; C>T)/β^E^
2	M	7.9	1925.8	β^0^(codon 17; A>T)/β^E^
3	M	2.5	2099.7	β^0^(codon 41/42; -TTCT)/β^E^
4	F	9.0	2624.6	β^0^(IVS2-654; C>T)/β^0^(IVS2-654; C>T)

To isolate CD34^+^ cells, a fraction of mononuclear cells was first separated from bone marrow samples or leukapheresis products using IsoPrep density-gradient centrifugation according to the manufacturer’s protocol (Robbins Scientific Corporation, USA). CD34^+^ cells were then separated from the fraction using magnetic bead selection (Miltenyi Biotech, Germany) following the manufacturer’s instruction. The percent purity of selected CD34^+^ cells, determined using flow cytometry (BD FACverse, BD Biosciences, US), was greater than 85%.

Isolated CD34^+^ cells were next differentiated *in vitro* following a three-stage erythroid culture using the protocol from the Anstee laboratory, with modification [13]. In brief, cells were seeded in 6-well plates at the density of 2 × 10^5^ cells/mL in primary medium (IMDM (Biochrom, UK) containing 3% (v/v) human serum type AB (Sigma-Aldrich, UK), 10 μg/mL insulin (Tocris, US), 3 U/mL EPO, 1 ng/mL IL-3 (all from Peprotech, US), 10 ng/mL stem cell factor (SCF), 200 μg/mL transferrin (all from R&D systems, US), 2% (v/v) fetal bovine serum, 3 U/mL heparin, and 1% (v/v) penicillin/streptomycin (all from Merck, US). At day 8, cells were transferred to secondary medium (primary medium supplemented with 1000 μg/mL transferrin but without IL-3), and maintained at cell densities ranging between 1 to 3 × 10^6^ cells/mL. At day 11, cells were transferred to tertiary medium (primary medium with 1000 μg/mL transferrin without IL-3 or SCF), and further cultivated until day 18. Medium changes were performed twice a week. Cells were incubated at 37°C in a humidified atmosphere containing 5% CO_2_. On day 4, 7, 11, 14 and 18, cell viability was assessed using the Trypan blue (Sigma, UK) exclusion assay and cell morphology was monitored using Giemsa-stained cytospin preparations (Sigma, UK).

### Imaging flow cytometry

Cells were incubated with antibodies for cell surface markers including anti-GlyA-APC/Vio770, anti-CD36-PE, and anti-CD71-APC (1:20, 130100268, 130095472, and 130091727, respectively, all from Miltenyi Biotech, Germany). For intracellular staining, cells were fixed with 4% paraformaldehyde and resuspended in permeabilization buffer (Merck, US). Fixed cells were then incubated with anti-FoxO3 antibody (1:400, 12829, Cell Signaling Technology, US) for 1 h. After washing, cells were stained with FITC-conjugated secondary antibody (1:100, AP307F, Merck, US) for 30 min, and finally counter-stained with propidium iodide (1:50, Miltenyi Biotech, Germany) for 10 min. All incubation steps were performed at 4°C. Cells were analyzed using a Flowsight imaging flow cytometer (Merck, US) and images were analyzed with IDEAS 6.2 analysis software.

To determine levels of apoptosis following staining with cell surface markers, cells were incubated with Annexin V-FITC (1:50, Biolegend, US) and fluorescent intensities were measured using imaging flow cytometry. For the detection of ROS formation, cells were incubated for 30 minutes at 37°C with 10 μM CellROX green reagent (Invitrogen, US). Thereafter, cells were stained for cell surface markers, and analyzed using imaging flow cytometry.

### Immunoblotting

Cell lysates were collected in RIPA buffer (Merck, US) supplemented with phosphatase and protease inhibitors. Total cell protein concentration was quantified using the Pierce BCA protein assay kit (Thermo Fisher Scientific, US), according to the manufacturer’s protocol. Protein samples were separated by SDS-PAGE and transferred onto PVDF membranes. Membranes were blocked in 5% non-fat milk for 1 h, incubated with antibodies against phosphorylated AKT (Ser473) (1:1000, 051003, Merck, US), total AKT (1:1000, 9272, Cell Signaling Technology, US), phosphorylated FoxO3 (Ser253) (1:1000, 9466, Cell Signaling Technology, US), total FoxO3 (1:1000, 12829, Cell Signaling Technology, US), and β-actin (1:5000, ab6276, Abcam, US) overnight at 4°C, and then washed three times with TBST. The membranes were then incubated with horseradish peroxidase-conjugated secondary antibodies for 1 h at room temperature. Luminata Crescendo western HRP substrate (Merck, US) was used for detection.

### Quantitative real-time RT-PCR

Total RNA was extracted using the RNeasy plus mini kit (Qiagen, US) following the manufacturer’s instruction. cDNA was generated from total RNA using First-Strand cDNA synthesis kit (Invitrogen, US). Real-time PCR was conducted with a CFX96 PCR system (Biorad, US) using Luminaris color HiGreen qPCR master mix (Thermo Fisher Scientific, US) according to the manufacturer’s protocol. The data were normalized to GAPDH levels. All gene-specific primers used in this study are listed in S1 File.

### Statistical analyses

The data are expressed as means ± standard error of mean (SEM) from at least three independent experiments. Data between groups were compared using unpaired Student’s t-test or one-way analysis of variance (ANOVA), where appropriate. Statistically significant differences were considered at *P*-value of less than 0.05 (*) or less than 0.01 (**).

## Results

### *In vitro* differentiation of CD34^+^ cells from patients with β-thalassemia major recapitulates ineffective erythropoiesis

**Fig 1:**
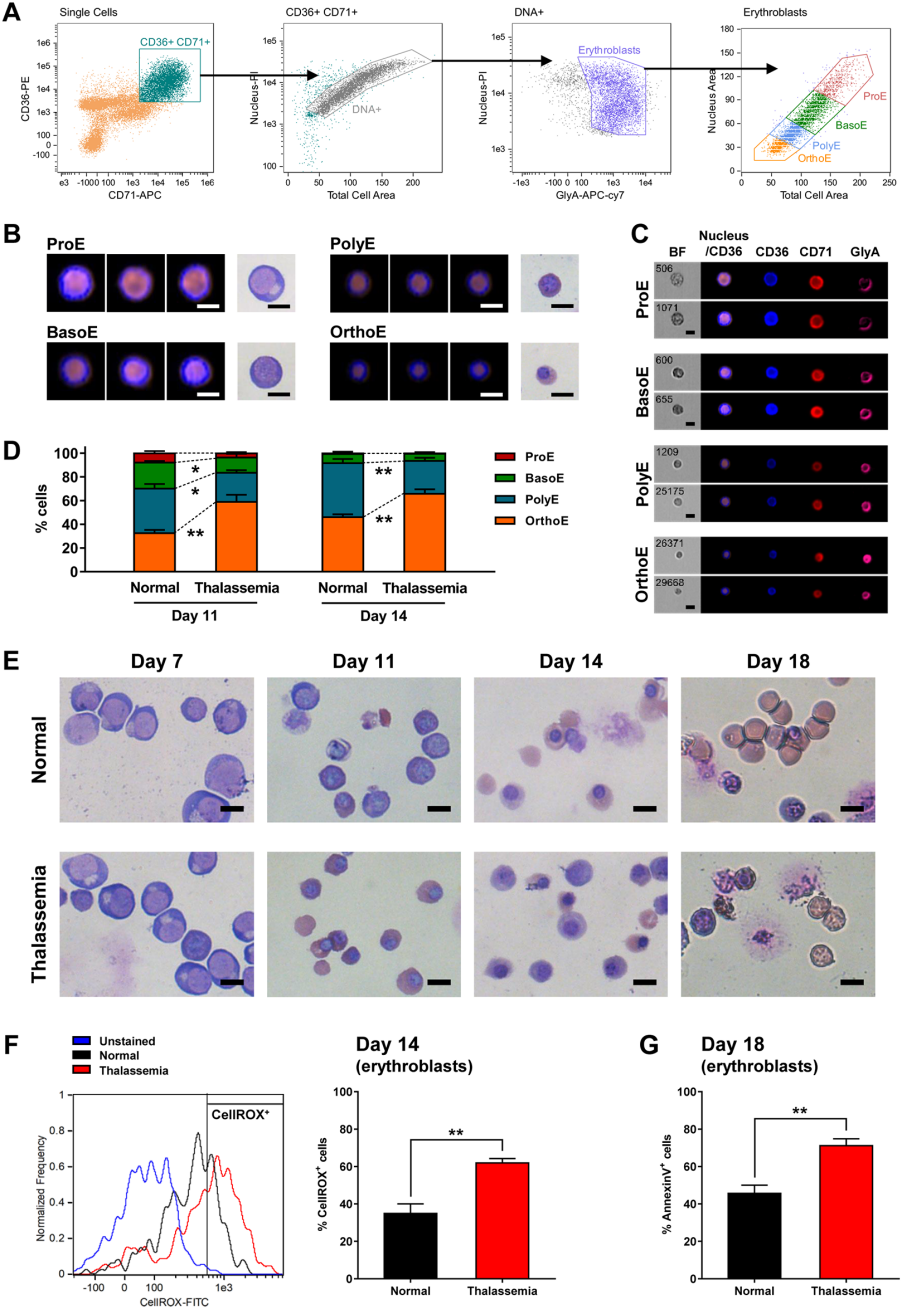
*In vitro* differentiation of CD34^+^ cells towards erythrocytes recapitulates ineffective erythropoiesis seen in β-thalassemia. (A) Imaging flow cytometry gating strategy for identifying each distinct stage of erythroblast populations. (B) Morphology of each stage of erythroblasts analyzed by imaging flow cytometry (merged fluorescence images of CD36 (blue) and propidium iodide (orange) representing cytoplasm and nucleus areas respectively), compared with the morphology observed in Giemsa-stained cytospin preparations (brightfield images). (C) Immunofluorescence images of erythroid cell surface markers CD36 (blue), CD71 (red) and GlyA (pink) in each stage of erythroblasts captured using an imaging flow cytometer. (D) Percentage of each stage of erythroblasts in normal and β-thalassemia cell populations on day 11 and 14, quantitated with imaging flow cytometry analysis. (E) Morphology of normal and β-thalassemia maturing erythroid cells at different days observed by light microscopy after Giemsa staining. (F) Imaging flow cytometry analysis of ROS levels in normal and β-thalassemia erythroblast populations on day 14 detected by staining cells with CellROX green (left panel), and percentage of CellROX-positive cells (right panel). (G) Percentage of Annexin V positive erythroblasts in normal and β-thalassemia samples on the final day of culture (day 18). The data are shown as means ± SEM. *P < 0.05, **P < 0.01 by Student’s t test.

To investigate FoxO3 regulation during erythroid differentiation in β-thalassemia, we developed an *in vitro* differentiation model using human CD34^+^ cells from patients with β-thalassemia major and normal donors. Isolated CD34^+^ cells were cultured in differentiation medium for 18 days. On day 11 and 14 of culture, the stage of erythroblast differentiation was classified using imaging flow cytometry, based on the levels of cell surface markers including CD36, CD71 and Glycophorin A (GlyA), as well as morphological characteristics as shown in Fig 1A–1C. In both normal and β-thalassemia cell populations on day 11, all developmental stages of erythroblasts including proerythroblasts (ProE), basophilic erythroblasts (BasoE), polychromatic erythroblasts (PolyE) and orthochromatic erythroblasts (OrthoE) were observed with noticeably different proportions of each subpopulation (Fig 1D). A significantly higher fraction of OrthoE was observed in in β-thalassemia samples on day 11 compared to normal groups (*P* = 0.00680) indicating accelerated erythroid differentiation, whereas BasoE and PolyE were a major subpopulation in normal control group. At day 14, erythroid cells in both two groups had predominantly reached terminal stages of erythroblast differentiation, PolyE and OrthoE. Giemsa-stained cytospins showed similar morphology of ProE in both normal and β-thalassemia specimens on day 7, but aberrant erythroblasts and enucleated cells with membrane defects were readily observed by day 18 only in the β-thalassemia group (Fig 1E). To detect ROS levels in the late erythroblasts, cells on day 14 were labeled with the fluorescent ROS indicator (CellROX green). A significant increase in CellROX positive cells was observed in the β-thalassemia group (61.93 ± 2.31%) relative to normal (34.93 ± 5.09%; *P* = 0.0029, Fig 1F). In addition to the increased oxidative stress, increased levels of apoptosis were detected in the β-thalassemia erythroid cell population using the Annexin V assay (71.14 ± 3.69% in β-thalassemia vs 45.66 ± 4.40% in normal; *P* = 0.0012, Fig 1G). Taken together, these results confirm that the culture system is capable of recapitulating the ineffective erythropoiesis seen in β-thalassemia. Single-cell analysis using imaging flow cytometry was also shown to be an effective methodology for staging the human erythroblasts during the *in vitro* differentiation.

### Normal erythroblasts maintain high activity of FoxO3 during differentiation

**Fig 2:**
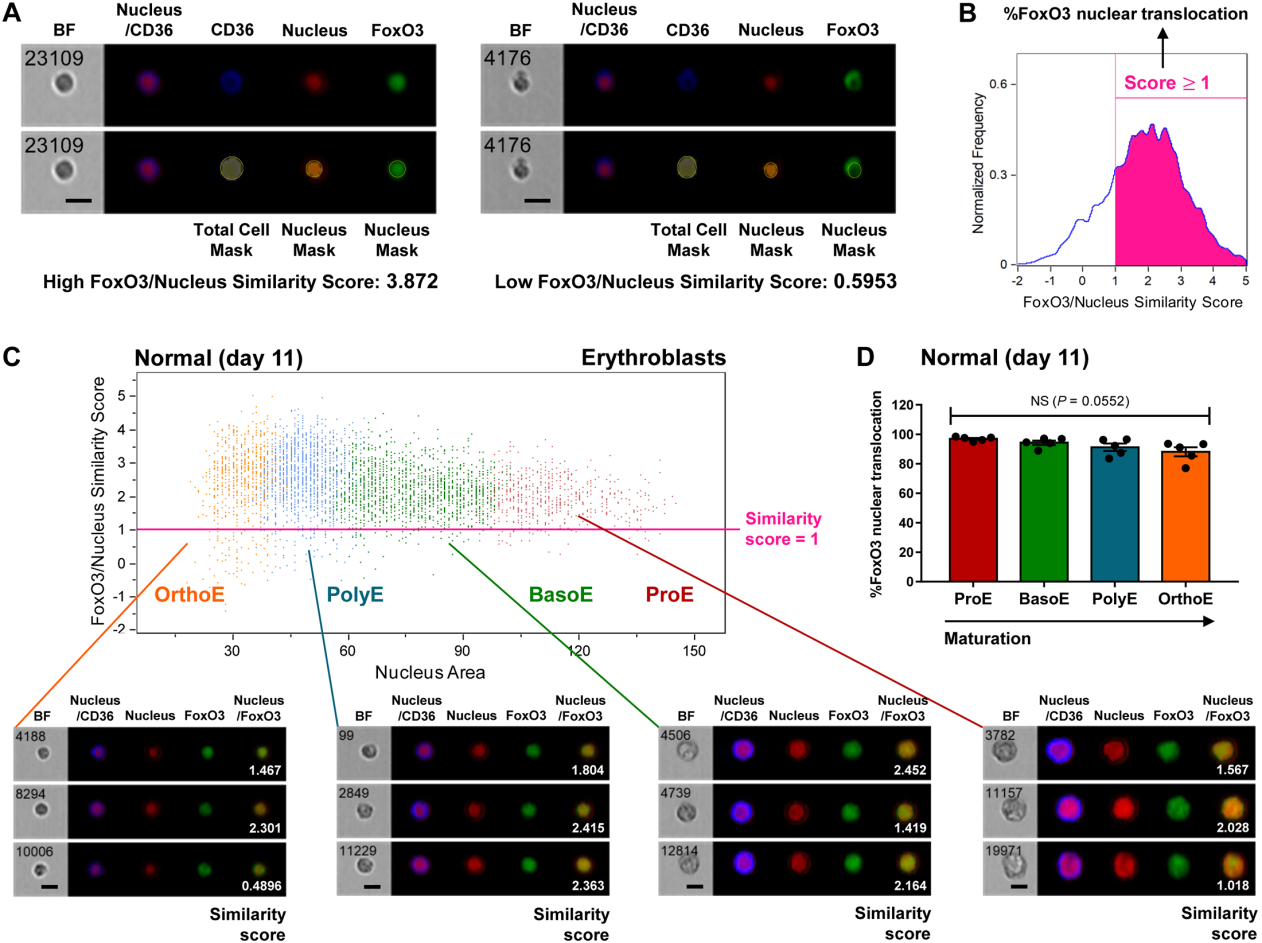
Normal erythroblasts display high levels of FoxO3 nuclear translocation. (A) Representative imaging flow cytometry analysis of FoxO3/nucleus similarity scores. Total cell mask and nucleus mask were monitored based on CD36 and PI signals, respectively. The degree of similarity between the images of nucleus and FoxO3, the FoxO3/nucleus similarity score, were used to determine the levels of FoxO3 nuclear localization. (B) Representative quantification of percent FoxO3 nuclear translocation from the cell population based on FoxO3/nucleus similarity scores. (C) The similarity scores of normal erythroblasts observed on day 11 shown with brightfield (BF) images, fluorescence images, and similarity scores (white numbers). Fluorescence data show the following markers: merged images of propidium iodide (red) and CD36 (blue) representing cell morphology; images of propidium iodide; images of FoxO3 (green); and merged images of nucleus and FoxO3. (D) % FoxO3 nuclear translocation of each stage of normal erythroblasts on day 11. The data are presented as means ± SEM (n = 5 independent experiments).

Since FoxO3 is known to be an important player in erythropoiesis, we used a high-throughput approach to quantify the FoxO3 activity in individual erythroblasts at all stages of differentiation (Fig 2). Cells on day 11 of differentiation, which had predominantly reached the erythroblast stage, were collected for the analysis. FoxO3 activity was determined based on the similarity score between the nuclear marker and the immunostaining of FoxO3 using the IDEAS analysis software (Fig 2A). Cells with a similarity score higher than 1 (cells with nuclear localized FoxO3) were counted as positive for FoxO3 activity (Fig 2B). The majority of normal erythroblasts exhibited high FoxO3/nucleus similarity scores throughout erythroblast maturation (Fig 2C). There was no statistically significant difference in the percent of FoxO3 nuclear translocation from distinct maturation stages of normal erythroblasts (*P* = 0.0552) (Fig 2D). Immunofluorescence images were also acquired using imaging flow cytometry and confirmed that FoxO3 remained localized to the nucleus during all stages of differentiation in normal erythroblast development.

### β-Thalassemia cells show decreased activity of FoxO3 during late erythroblast maturation

**Fig 3:**
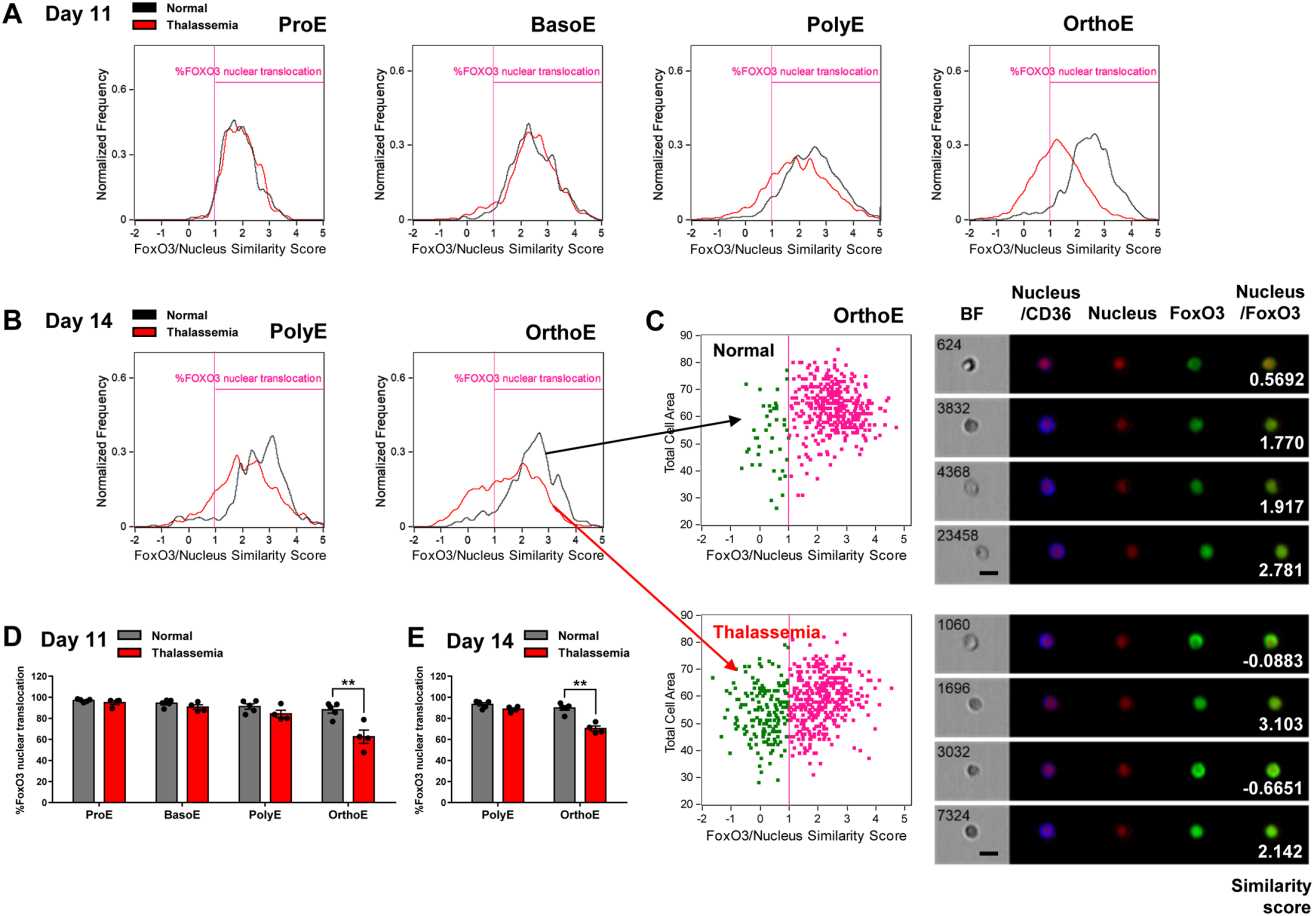
Decreased FoxO3 nuclear localization during late erythroblast maturation in β-thalassemia. Imaging flow cytometry analysis with FoxO3/nucleus similarity scores of each stage of erythroblasts from normal and β-thalassemia samples on day 11 (A) and day 14 (B). (C) Analysis of similarity scores of OrthoE in normal and β-thalassemia on day 14 with representative brightfield (BF) images, fluorescence images, and similarity scores (white numbers). Summary of % FoxO3 nuclear translocation in each stage of normal and β-thalassemia erythroblasts on day 11 (D) and day 14 (E). The data from normal cells on day 11 presented in Figs 3A and D were based on those in Fig 2D. Data are expressed as means ± SEM. **P < 0.01 was calculated using the Student’s t test. Normal group, n = 5 independent experiments (from 5 different normal subjects); β-thalassemia group, n = 4 independent experiments (from 4 different patients).

To explore how the FoxO3 activity was modulated in β-thalassemia, we next compared the activity of FoxO3 in the β-thalassemia erythroblasts at different developmental stages with the normal erythroblasts. On day 11, there was no significant difference in the fraction of cells with positive FoxO3 activity between normal and β-thalassemia cells in the ProE and BasoE subpopulations (Fig 3A and 3D). A slight reduction of FoxO3 activity was observed in the PolyE subpopulation at day 11. However, reduced FoxO3 activity was most pronounced in the OrthoE subpopulation (63.02 ± 5.33% in β-thalassemia vs 88.10 ± 3.12% in normal; P = 0.0036, Fig 3A and 3D). The analysis of FoxO3/nucleus similarity in those cells was performed at single cells as shown in Fig 3C. Differences in FoxO3 activity between normal and β-thalassemia in the OrthoE subpopultion could be observed through day 14 (67.87 ± 4.69% in β-thalassemia vs 89.81 ± 2.12% in normal; P = 0.0027) (Fig 3B and 3E). Collectively, these results infer that FoxO3 remains active during early erythroblast differentiation, in both the normal and the β-thalassemia cells. In contrast, FoxO3 activity diminishes in the β-thalassemia OrthoE cells at the late stage of erythropoiesis.

### β-Thalassemia cells exhibit activation of AKT and a corresponding decrease in FoxO3 target genes during late erythroid differentiation

**Fig 4:**
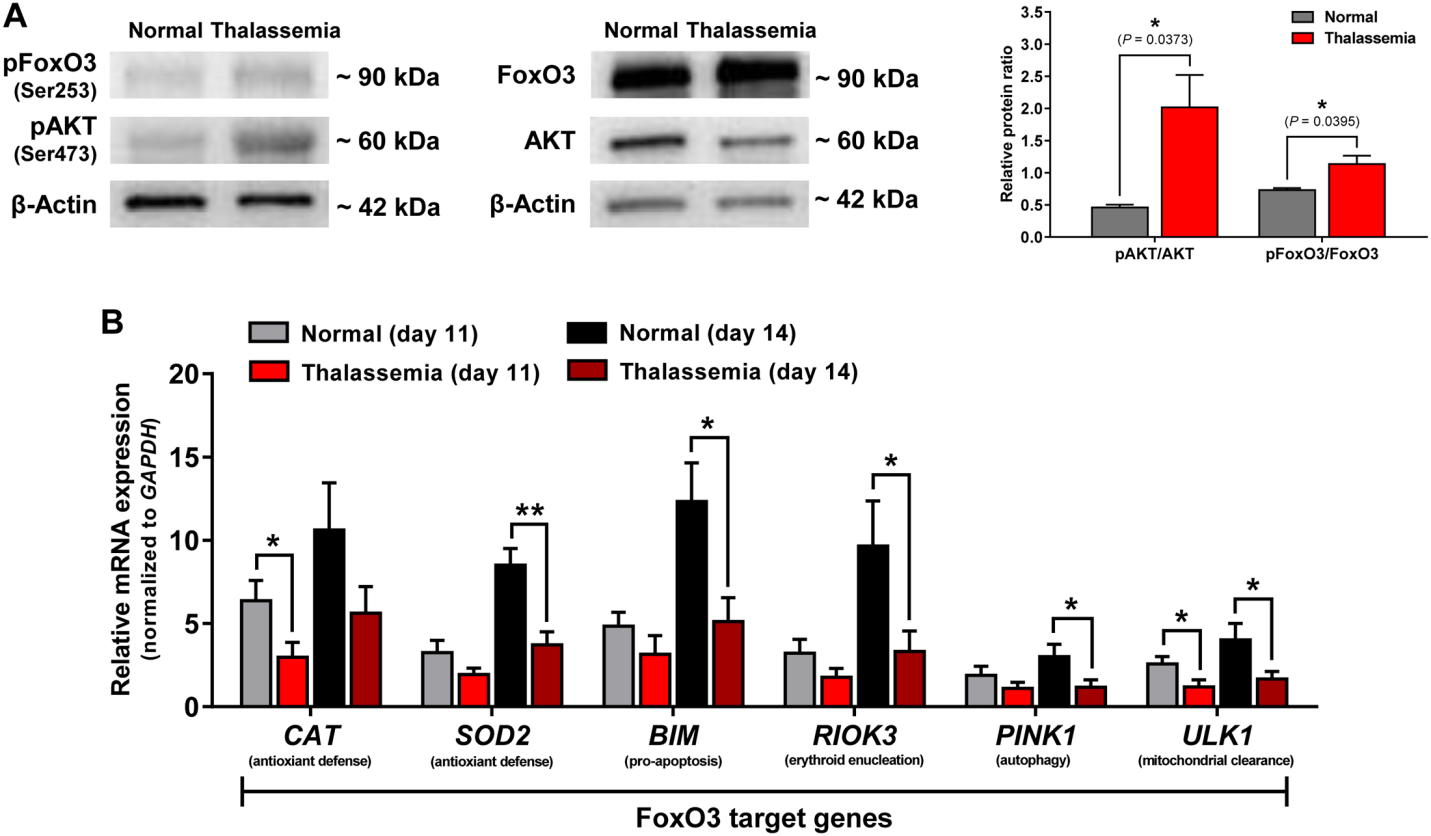
β-Thalassemia cells shows higher levels of phosphorylated AKT and FoxO3 and exhibit lower expression of FoxO3 target genes in late erythroid differentiation. (A) Expression of pAKT, total AKT, pFoxO3, and total FoxO3 in cultured cells on day 14 from normal and β-thalassemia groups analyzed by immunoblotting. β-Actin used as a protein loading control. Quantification of relative pAKT and pFoxO3 levels (Right panel). The data are presented as pAKT/AKT or pFoxO3/FoxO3 ratios and shown as means ± SEM. *P < 0.05, n = 3 independent experiments. (B) Relative mRNA expression levels of *CAT*, *SOD2*, *BIM*, *RIOK3*, *PINK1*, and *ULK1* in normal and β-thalassemia on day 11 and day 14 evaluated by real-time RT-PCR. *GAPDH* used as a reference gene. Data are expressed as means ± SEM. *P < 0.05, **P < 0.01 by Student’s t test.

Our studies demonstrated that FoxO3 nuclear localization was diminished during the late stages of β-thalassemia erythroblasts; however, the mechanistic cause of the diminished FoxO3 activity was not resolved. To better understand the cause of reduced FoxO3 activity, we investigated signaling pathways that could contribute to FoxO3 activation. FoxO3 nuclear-to-cytosolic localization can be altered by activation of the AKT pathway [11]. We first compared the levels of phosphorylated AKT (pAKT) (Ser473) and FoxO3 phosphorylation (pFoxO3) at an AKT specific site (Ser253) between normal and β-thalassemia cells at day 14 which were predominantly composed of cells in PolyE and OrthoE stages (Fig 4A). The relative phosphorylation level of AKT at Ser473 (pAKT) as well as phosphorylation of FoxO3 at Ser253 (pFoxO3) were observed to be significantly higher in β-thalassemia cells compared to normal cells. Using cells from day 14, we also measured the relative expression of FoxO3 target genes from both the normal and β-thalassemia groups (Fig 4B). The expression of *SOD2* (manganese superoxide dismutase), *BIM* (BCL-2-like 11), *RIOK3* (RIO kinase 3), *PINK1* (PTEN-induced putative kinase 1) and *ULK1* were prominently elevated from day 11 to day 14 in the normal cells and were significantly higher than in the β-thalassemia group. Altogether, activation of the AKT pathway appears to be a potential underlying cause of the reduced FoxO3a activity during late-stage erythropoiesis in β-thalassemia. The late erythroblasts derived from β-thalassemia cells showed diminished expression of FoxO3 target genes that function to counteract ROS formation, consistent with our previous observation of elevated ROS accumulation.

## Discussion

The prior investigation of FoxO3 activity during erythropoiesis indicated essential roles for this protein in several steps including maintenance of the hematopoietic stem cell pool [14, 15], cell cycle progression in the early-stage erythroblasts [16], and erythroid maturation in the late-stage erythroblasts [7]. Previous studies using murine models demonstrated the significance of FoxO3 for normal erythropoiesis [16] but the dynamics of FoxO3 activity in human cells especially during ineffective erythropoiesis in severe β-thalassemia have not been elucidated. A major hurdle preventing this analysis was the heterogeneity of erythroid cell populations [17], making it difficult to monitor changes of regulatory proteins by using conventional end-point assays or low-throughput techniques. In this study, we successfully utilized imaging flow cytometry to help quantitate the localization of FoxO3 from the individual erythroid cells at different developmental stages in both normal and β-thalassemia cells. Interestingly, our technique reveals that FoxO3 remains active throughout normal erythroblast maturation, while its activity drops significantly during the terminal stage of erythropoiesis in β-thalassemia cells, specifically in the OrthoE subpopulation.

In agreement with the diminished FoxO3 activity, we found that the transcriptional expression of FoxO3 target genes was substantially lowered in the β-thalassemia cells, especially for *SOD2*, *PINK1*, *ULK1* and *RIOK3* which were formerly identified to be responsible for ROS defense, autophagy, mitochondrial clearance and erythroid enucleation, respectively [7, 8]. Earlier studies confirmed that the activities of the listed enzymes are pivotal for terminal erythroid maturation [7, 18, 19]. In particular, the antioxidant enzymes are required to handle the elevated levels of ROS that occur during hemoglobin synthesis [12, 20–22]. Taken together, these findings support the role for loss of FoxO3 activity as one of the attended causes of dysregulated erythropoiesis in the β-thalassemia cells.

Consistent with previous reports, our study has revealed that the level of pAKT is significantly increased in β-thalassemia erythroblasts as compared to normal cells. The elevated phosphorylation of AKT has been suggested to be triggered by increased ROS formation. The rationale for this idea is due to the fact that oxidative stress can promote glycolysis, the TCA cycle, and oxidative phosphorylation, which in turn will elevate intracellular levels of Ca^2+^ and ATP, eventually leading to the activation of AKT [23]. Mechanistically, we propose that the elevated ROS levels in β-thalassemia cells may play an important role in the activation of AKT, potentially resulting in the repression of FoxO3 activity and reducing the response to oxidative stress. Several dysregulated pathways in β-thalassemia, such as the apoptotic signaling cascade, are also thought to be stimulated by ROS. However, a direct link between increased ROS and the activation of AKT has not been thoroughly examined [5]. In addition to AKT, modulation of FoxO3 is also involved in other pathways through regulation of JNK, MST1, ERK, and SIRT1 [11, 24, 25]. Consequently, further analysis is needed to fully establish the exact mechanism leading to changes of FoxO3 activity in β-thalassemia.

The use of potential therapeutic agents targeting FoxO3 in the treatment of β-thalassemia is currently under investigation [12]. Resveratrol, a compound that was previously found to activate FoxO3, can reduce the levels of pAKT and increase anti-oxidant enzymes in cultured erythroid cells derived from patients with β-thalassemia intermedia. Moreover, resveratrol treatment could enhance erythropoiesis in β-thalassemic mice [6]. However, a mechanistic understanding of the mode of action of resveratrol as well as its efficacy and safety is needed before continuing to the next phase. This is a significant challenge in the development of novel effective drugs for the treatment of β-thalassemia. Our study here may provide a useful tool for tracking FoxO3 activity during erythropoiesis and could be utilized in the investigation of potential compounds in the drug development process.

Erythropoiesis is dynamically orchestrated by multiple genes and regulatory proteins [26–28]. Although the activation of FoxO3 may ameliorate the ineffective erythropoiesis [6], in practice dysregulation of several proteins and pathways simultaneously exacerbates the pathophysiology of β-thalassemia. This dysregulation of several pathways leads multiple effects in β-thalassemia cells including premature apoptosis, uncontrolled expansion of early erythroid progenitors, accelerated differentiation, and arrested terminal erythroid maturation [5]. Therefore, therapeutic combinations targeting key regulators that contribute to ineffective erythropoiesis may be a potential strategy for β-thalassemia treatment. In this study the FoxO3 activity profile has been evaluated in common genotypes of severe β-thalassemia. However, several different variants and disease severity levels are found in β-thalassemia making it likely that different FoxO3 regulation patterns as well as other pathophysiological mechanisms are evident among patients. Hence, the ineffective erythropoiesis and relevant regulatory pathways should be further examined in other subtypes of β-thalassemia with different disease severity and other disease-modifying factors. Such knowledge would empower the development and validation of enhanced personalized care for β-thalassemia patients, especially for pharmacological therapy and gene therapy approaches.

## Conclusion

In summary, this study demonstrates a high-throughput approach to quantitatively evaluate FoxO3 localization at the single-cell level from heterogeneous erythroid cell populations. The demonstration of the FoxO3 nuclear translocation profile in the late-stage erythroblasts presented here may be a key to exploring the regulation of FoxO3 during terminal erythropoiesis in severe β-thalassemia. Better understanding of the mechanisms underlying FoxO3 activity has the potential to explain the ineffective erythropoiesis seen in β-thalassemia and the pathophysiology of this disease.

## Supporting information

S1 File Primer sets for real-time RT-PCR analysis. (PDF)
