# Supplementary material for: Investigation of FoxO3 dynamics during erythroblast development in β-thalassemia major
Source: PLoS One. 2017 Nov 3;12(11):e0187610. doi: 10.1371/journal.pone.0187610 (PMC5669432; doi:10.1371/journal.pone.0187610)
Supplement: S1 File — (PDF) [file pone.0187610.s001.pdf]

Primer sets for real-time RT-PCR analysis

Gene	Forward sequence	Reverse sequence	Amplicon (bp)	Accession number
BIM	TGGCAAAGCAACCTTCTGATG	GCAGGCTGCAATTGTCTACCT	64	NM_001204113
CAT	GAACTGTCCCTACCGTGCTCGA	CCAGAATATTGGATGCTGTGCTCCAGG	156	NM_001752
GAPDH	GAAGGCTGGGGCTCATTT	CAGGAGGCATTGCTGATGAT	138	NM_001289746
PINK1	TACCAGTGCACCAGGAGAAG	GCTTGGGACCTCTCTTGGAT	209	NM_032409
RIOK3	TGTGGCATGCTGGAAAGGTCTG	GCTTCCTTGACTCCTCCTTTCTGG	136	NM_003831
SOD2	ACCTCAGCCCTAACGGTGGT	CAGCCGTCAGCTTCTCCTTAAA	102	NM_001322820
ULK1	TCGAGTTCTCCCGCAAGG	CGTCTGAGACTTGGCGAGGT	134	NM_003565
